# Haematological and biochemical reference values for Bonelli’s eagles in the wild and in captivity: implications for conservation and rehabilitation programs

**DOI:** 10.1186/s12917-025-04790-0

**Published:** 2025-05-15

**Authors:** Fernando González, Octavio Perez-Luzardo, Luis Revuelta, Laura Suárez-Regalado, Virginia Moraleda-Fernández, Alicia Carrero-Ruíz, Laura Del Río

**Affiliations:** 1 Grupo de Rehabilitación de la Fauna Autóctona y su Hábitat (GREFA), Monte del Pilar, Majadahonda, 28220 Madrid, Spain; 2 Sección Departamental de Farmacología y Toxicología, Facultad de Veterinaria, Universidad Complutense de Madrid, 28040 Madrid, Spain; 3 Unidad de Toxicología, Instituto Universitario de Investigaciones Biomédicas y Sanitarias (IUIBS), Universidad de Las Palmas de Gran Canaria, 35016 Las Palmas de Gran Canaria, Spain; 4 Sección Departamental de Fisiología Veterinaria, Facultad de Veterinaria, Universidad Complutense de Madrid, 28040 Madrid, Spain; 5 Sección Departamental de Anatomía y Embriología, Facultad de Veterinaria, Universidad Complutense de Madrid, 28040 Madrid, Spain; 6 Departamento de Sanidad Animal, Facultad de Veterinaria, Regional Campus of International Excellence Campus Mare Nostrum, University of Murcia, 30100 Murcia, Spain; 7 Grupo de Estudio de Medicina y Conservación de los Animales Salvajes (GEMAS), Madrid, Spain

**Keywords:** Reference values, Blood chemistry, Haematology, Bonelli’s eagle, Aquila fasciata, PCA

## Abstract

**Background:**

Haematological and clinical biochemistry analyses are essential tools for evaluating the health status of avian species, including the endangered Bonelli’s eagle. Despite their importance, existing reference intervals (RIs) for such parameters in raptor species are frequently constrained by limited sample sizes, thus complicating clinical interpretations. This research followed the American Society for Veterinary Clinical Pathology (ASVCP) guidelines to establish haematological and clinical biochemistry reference intervals for the Bonelli’s eagle, utilizing an indirect approach. Conservation initiatives of the Bonelli’s eagle have provided a unique opportunity to gather a high number of samples under various conditions, facilitating a comprehensive comparison between wild and captive populations. Over the course of six years, from 2016 to 2021, our research analyzed 516 blood samples collected within the framework of a European Life Project. After rigorous data cleaning and stringent selection criteria application, a representative sample of 184 birds was determined. Various physiological parameters and blood lead levels were quantified in healthy individuals. Additionally, we used Principal Component Analysis to discern distinctions between populations and to investigate potential interrelationships among the diverse parameters.

**Results:**

We established Reference Intervals for three distinct reference groups of birds: wild nestlings ($n=72$), captive nestlings ($n=45$), and adults ($n=49$). Our findings indicate that age significantly affects many blood parameters. Meanwhile, gender impacts only a few parameters in adult birds. Notably, wild nestlings exhibited higher levels of AST, K, TP, and Pb in their blood compared to those bred in captivity.

**Conclusions:**

This paper provides the first reliable RI for physiological, haematological, and clinical biochemistry parameters in both nestling and adult Bonelli’s eagles. The data will augment the knowledge of the physiology of this endangered raptor, contributing significantly to the understanding and monitoring of both free-living birds and captivity programs.

## Background

Haematology and clinical biochemistry are instrumental in assessing the health status of raptors, including endangered species. By facilitating the early detection of subclinical processes, blood parameters provide crucial insights into bird health, as avian species rarely exhibit overt clinical signs until disease progression is significantly advanced [1, 2]. They are useful for both clinical management in captivity and rehabilitation centres, and for monitoring wild populations and translocation programmes [3, 4]. Furthermore, owing to their position atop the food chain, raptors like the Bonelli’s eagle (*Aquila fasciata*) serve as excellent indicators of ecosystem health, as any alterations in their blood parameters may reflect changes in their habitat, such as fluctuations in food availability, exposure to pathogens, and environmental toxins [5]. Given the high prevalence of lead (Pb) exposure in raptors and its severe effects on certain species, it is important to also, determine Pb levels, alongside standard biochemical and hematological markers. The Bonelli’s eagle is a large raptor belonging to the order Accipitriformes. It has a wide but fragmented distribution across the Iberian Peninsula, North Africa, China, and several Indonesian islands in Southeastern Asia. The current estimated population of breeding pairs in Europe is approximately 1100-1200, with 80% of them residing in Spain with a wide distribution [6, 7]. However, this species is experiencing a significant decline throughout its entire range, of about 30% over the past 54 years [8, 9]. In recent years, multiple initiatives have been launched for the conservation of the Bonelli’s eagle, including two consecutive European Life Projects (ELP). These initiatives involve the reintroduction of captive-bred specimens into areas where the species was recently extinct [10, 11]. Both *ex-situ* (captive breeding) and *in-situ* (in the natural habitat) initiatives included the annual examinations of eagles, assessing their health status prior to the breeding season. This has provided a unique opportunity to sample a large number of birds, to obtain reliable reference intervals (RIs) for this endangered species under different conditions, and evaluate the potential effects of *ex-situ* management by comparing wild and captive birds.

Blood reference intervals are available for many raptor species, but are often derived from small sample sizes, complicating the interpretation of whether a specific blood parameter measurement reflects a true pathological condition or a normal variation associated with stress, age, sex or other physiological factors at the time of sampling [1, 12, 13]. The American Society for Veterinary Clinical Pathology (ASVCP) recommends a comprehensive understanding of the reference population’s demographics and a careful examination and elimination of potential sources of biological and laboratory variability when developing reference intervals (RIs) [14]. Regrettably, to date there are limited haematological and clinical biochemistry values for the Bonelli’s eagle, and the published data should be interpreted cautiously, given the small sample size and non-standardized conditions under which the ranges were obtained [15].

In this paper we provide reference intervals for some physiological parameters as well as haematology, clinical biochemistry and Pb values in nestlings and adult Bonelli’s eagles. Understanding the baseline levels of Pb is particularly important, as susceptibility appears to differ between species [16]. To our knowledge, this is the first study to determine reliable RIs for this endangered species, following the recommendations of the ASVCP, and examining the effects of sex, age and captivity on these parameters. The data will augment our knowledge of the physiology of this endangered raptor, contributing significantly to the better understanding and monitoring of both free-living birds and captivity programs.

## Methods

### Animals and database

The objective of this retrospective study was to establish haematological and clinical biochemistry reference intervals for Bonelli’s eagles, utilising samples obtained during the monitoring programmes of a ELP (Aquila-A-Life 16/NAT/ES/000235), designed to evaluate the species’ health status and assist in the revitalisation of its population in the Western Mediterranean. Blood sampling and monitoring of physiological parameters such as body temperature (in $^{\circ }$C), heart rate (beats per minute, bpm), and respiratory rate (breaths per minute, bm) were performed as part of annual health assessments. The data were systematically stored in the database of the Wildlife Rehabilitation Centre “Grupo de Rehabilitación de Fauna Autóctona y su Hábitat” (GREFA WRC) in Madrid, Spain.

**Fig. 1:**
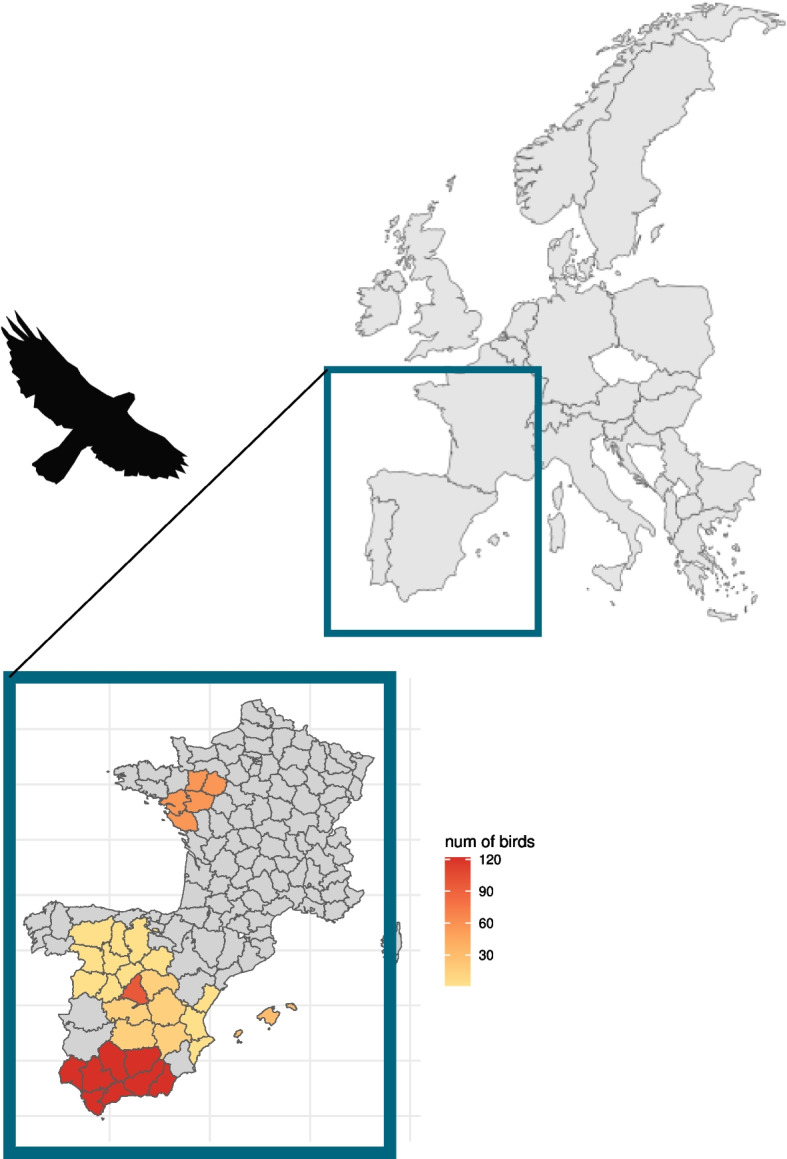
Geographic distribution of the bird origins involved in the study. Nest locations include Andalucía, Madrid, Balearic Islands, Castilla la Mancha, Valencia, and Castilla-León. Breeding centers are situated in Madrid and Vendee (France). Colors depict the number of birds sampled from each region

The study incorporated haematological and clinical biochemistry results from a diverse cohort of 322 Bonelli’s eagles (see Table 1), from blood samples collected between January 2016 and December 2021 and analysed at the GREFA WRC’s laboratory. The geographical origins of the studied avian subjects are explicitly detailed in Fig. 1.

**Table 1:** Number and geographic origin of birds incorporated in the study, sampled from January 2016 to June 2021

Region of sampling	Nestlings		Juveniles		Adults		Total
	Wild	Captive	Wild	Captive	Wild	Captive	
Madrid, (Spain)^a^	12	18	2	7	9	49	**97**
Andalucía, (Spain)	122						**122**
Vendee, (France)		56					**56**
Balearic Islands, (Spain)	29						**29**
Castilla la Mancha, (Spain)	15						**15**
Other regions (Spain)^b^	3						**3**
Total	**181**	**74**	**2**	**7**	**9**	**49**	**322**

^a^Birds were admitted to the GREFA WRC

^b^Other regions comprise Valencia ($n=2$) and Castilla-León ($n=1$)

Birds were classified according to age categories: nestlings (< 50 days old), juveniles (< 4 years old), and adults (> 4 years old). The wild bird sample subset included 181 nestlings collected from various regions across the Iberian Peninsula, along with blood samples from 2 juveniles and 9 adults admitted to the GREFA’s WRC. Table 1 also shows the geographic locations of the nests where the birds were sampled. Other bird samples were procured from 74 nestlings hatched either at the GREFA WRC ($n=18$) or at the Ligue pour la Protection des Oiseaux’s breeding centre in Vendee, France ($n=56$). Additionally, blood samples of 7 juveniles and 49 adults from the breeding programme were included. Captive Bonelli’s eagles resided under natural photoperiods in breeding enclosures measuring 7 metres high, 5 metres long, and 4 metres wide. Each enclosure was equipped with nesting platforms. The diet of the birds consisted of rat, rabbit, or quail, with water available *ad libitum*.

**Fig. 2:**
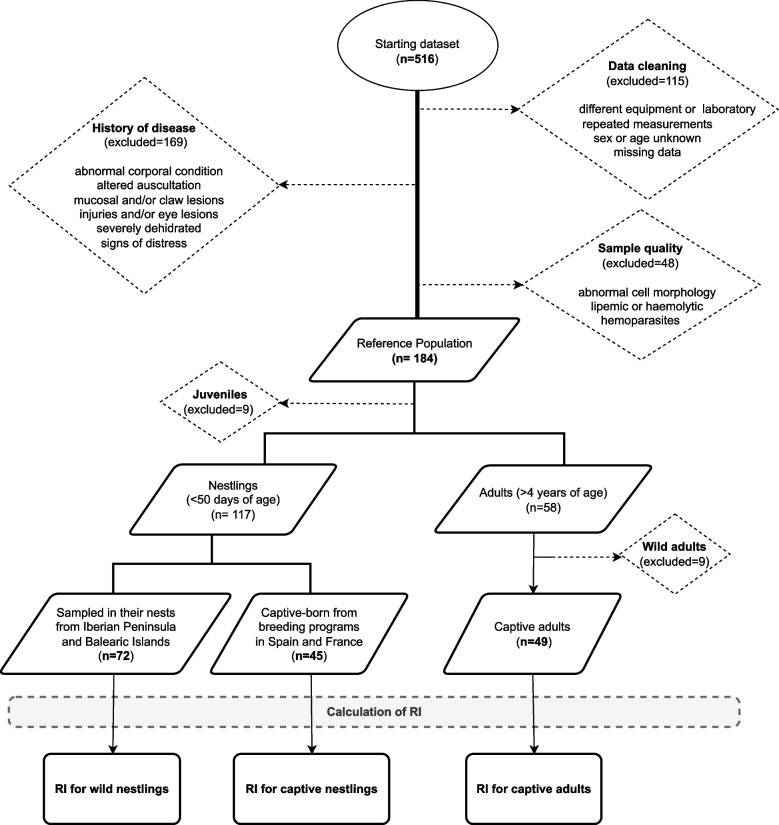
Schematic representation of study phases for the selection of reference populations. Outlined Circle: initial dataset; Dashed Diamond: application of exclusion criteria; Parallelogram: curated dataset; Dashed Rectangle: data analysis phase; Solid Rectangle: defined reference populations

The preliminary database contained 516 records, with several animals undergoing two or more analytical examinations. We used extensive clinical information recorded during routine veterinary physical examinations to exclude invalid records. To uphold data quality and integrity, data cleaning, profiling, validation, and cleansing processes were conducted to identify inconsistencies, duplicate entries, or missing values (Fig. 2). The specific selection criteria and rigorous exclusion parameters for samples and records are enumerated in Table 2. Consequently, the final dataset for RI computations consisted of records from 184 healthy birds.

**Table 2:** Selection and exclusion criteria for the reference population

Classification	Criteria	Categories included
Selection criteria	Age	Nestlings, Juveniles, Adults^a^
	Sex	Female, Male
	Corporal condition (cc)	Normal (cc=3)
	Season of sampling	Spring
	Laboratory of analysis	GREFA WRC
Exclusion criteria	Clinical	History of disease (by veterinary inspection)^b^
	Low cc or obesity	cc <3 or cc >4
	Elderly	Age >16 years
	Inadequate sample	Lipemic, haemolytic

^a^Nestlings are defined as $<50$ days of age, Juveniles $<4$ years of age, and Adults as $>4$ years of age

^b^A veterinary inspection entails a thorough physical examination and auscultation, recording of weight, temperature, respiratory rate, and heart rate, assessment of the integrity of oral, cloacal, and eye mucosas, evaluation of feather condition, signs of dehydration, trauma, presence of injuries, and identification of lesions indicative of *Trichomonas spp*. infection

### Blood sampling

Blood samples were procured from free-ranging and captive-born nestlings at their nests when they were from 6–7 weeks old. All animal handling was conducted under the supervision of a veterinarian, using specialised equipment such as hoods and towels to mitigate stress. Sample collection was carried out by experienced wildlife veterinarians using standard raptor handling techniques. For all birds, blood analyses were incorporated into their routine medical examinations. Health indicators were registered for each bird, including behavior, body condition (cc), weight, body temperature ($^{\circ }$C), heart rate (beats per minute, bpm), respiratory rate (breaths per minute, bm). In addition, cloacal, ocular and oral mucosa examinations, the assessment of claws and feathers integrity, as well as the presence of signs of dehydration, trauma, injuries, or *Trichomonas spp* infection symptoms were documented (Table 2). The sex of each bird was determined using molecular techniques, as previously described [17]. In brief, the employment of primers (P2 and P8) of the *chd* gene (Chromodomain helicase-DNA- Binding protein), a region conserved in both Z and W sex chromosomes in nearly all avian species, enabled us to differentiate females from males by detecting two fragments in females (heterogametic sex) and only one in males (homogametic sex). An additional primer (P8-MP) was used to enhance the resolution of fragment visualisation, with PCR products analysed via capillary electrophoresis using acrylamide polymers.

Blood was collected via venipuncture from the cubital vein using 23-gauge disposable needles and 2 ml plastic syringes. Following collection, a blood smear was immediately prepared from a drop of the whole blood. The remaining blood was divided into an ethylenediaminetetraacetic acid-K2 (EDTA) blood tube (Royal Blue Vacutainer Hemogard 1 Vacuum tubes, Beckton Dickinson) and stored at 4–6$^{\circ }$C for subsequent haematological and Pb analysis within 24 hours post-collection. Another aliquot of blood was placed in a lithium-heparin tube (1.8 mg/ml blood), centrifuged at 3500 rpm for 10 minutes to procure plasma for clinical biochemistry and proteinogram analysis. Plasma aliquots were stored at −20$^{\circ }$C until analysis. A small volume was transferred to a heparinized microcapillary tube for packed cell volume (PCV) determination. For Pb level detection, a third portion of the blood from each bird was stored at −20$^{\circ }$C and sent to the Laboratory of Toxicology, University of Las Palmas, Gran Canaria, Canary Islands, Spain. Detailed laboratory methodologies are described in the following section.

### Laboratory methods

Sample quality was appraised for each analysis, facilitating the identification and exclusion of records demonstrating abnormal cell morphology, presence of hemoparasites, or hemolytic or lipemic samples during the data cleansing process (Table 2).

Haematological investigation included haematocrit or PCV, Red blood cell count (RBC), haemoglobin level (Hb), mean cellular volume (MCV), mean corpuscular haemoglobin (MCH), and mean corpuscular haemoglobin concentration (MCHC), white blood cell count (WBC), and WBC differential count. PCV was ascertained utilizing standard hematocrit methodologies, post centrifugation in a microhematocrit centrifuge (Nahita microhematocrit centrifuge 2900) for 6 minutes at 11000 rpm. Hb concentration (*g*/*dL*) was estimated as a third of the PCV, in accordance with [18]. Additionally, in samples taken from 2019 to 2021, Hb was also determined using an automatic Hb analyzer (HemoCue Hb 201 System$^{\copyright }$).

Cell morphology and the presence of haemoparasites were also evaluated and registered for each sample. Total RBC and WBC were manually counted using the Natt-Herrick solution (1:200 dilution) and a Neubauer hemocytometer (Improved Neubauer Counting Chamber, Blaubran$^{\copyright }$), following protocols previously described [12, 19, 20]. Blood smears were stained with May-Grünwald-Giemsa stains, and leukocyte types were classified based on staining characteristics and morphology after examining 100 white blood cells on the blood film. The differential leukocyte count was obtained by multiplying the WBC by the percentage of each leukocyte type.

Plasma biochemistry parameters were quantified using the VetScan VS2, a serum clinical biochemistry analyzer (Abaxis, Inc.$^{\copyright }$, Zoetis, Madrid), using avian/reptilian-specific rotors (Avian/Reptilian Profile Plus rotor, Abaxis) in accordance with the manufacturer’s guidelines. The analytes measured included Albumin (Alb), Aspartate aminotransferase (AST), Calcium (Ca), Cholesterol (Cho), Creatine phosphokinase (CPK), Creatinine (Crea), Glucose (Glu), Phosphorus (Phos), Potassium (K), Sodium (Na), Total protein (TP), and Uric Acid (UA).

Blood Pb concentration was analysed using two distinct methods. Routinely, Pb was determined in GREFA’s WRC clinical laboratory within 24 hours of blood extraction, using anodic stripping voltammetry with an electrochemical system (LeadCare II Blood Lead Testing System$^{\copyright }$, ESA Inc, Chelmsford, MA, USA), adhering to the manufacturer’s instructions. As the majority of samples were near or below the detection limit (the analytical reporting range was 3–65 $\mu g/dL$ or 0.14–3.14 $\mu mol/L$), for a more accurate quantification, inductively-coupled plasma mass spectrometry (ICP-MS) was also performed (Agilent 7900 ICP-MS$^{\copyright }$). All data was recorded and analysed using the Agilent MassHunter Data Acquisition software 4.2 at the Laboratory of Toxicology, University of Las Palmas, Gran Canaria, Canary Islands, Spain, as previously described [21].

### Selection of reference individuals

The process of data analysis and establishing de novo Reference Intervals (RIs) for Bonelli’s eagles was conducted in compliance with the ASVCP guidelines as detailed in [14].

Subsequent to data cleansing and conversion to International Units for each parameter, outliers were identified and excluded employing Horn’s algorithm and Tukey’s interquartile fences. In datasets including zeros, Cook’s distance was used to pinpoint outliers.

Normality of each parameter was then evaluated using the Kolmogorov-Smirnov test for parameters with $n>50$, and the Shapiro-Wilk test for those with $n<50$. For RIs definition, if the reference population exceeded 40 samples, robust methods with 90% confidence intervals (CIs) with bootstrapping (for Gaussian symmetrically distributed data) or parametric analysis (Gaussian asymmetrical distribution) were implemented. For non-normally distributed data, non-parametric methods were utilised, and the mean plus-minus standard deviation (sd) and median were reported. When the reference population ranged between 20 and 40, normal data applied parametric methods, whereas non-normal data utilised robust methods with 90% CI, presenting minimum and maximum values. For parameters with less than 20 reference samples, or a dataset with a substantial number of zeros precluding RI calculation, only the mean value plus-minus standard deviation (SD), median and the range of observed values are presented. The number of data used for the calculation of RIs for each parameter are indicated in the tables.

### Statistical analysis

The impact of age, sex, and captivity on blood parameters was evaluated by univariant parametric or non-parametric tests (*t*-test or *U*-test), based on data normality and distribution. RIs are presented for the entire population only when no statistical differences between males and females were observed; otherwise, separate tables for each bird group were created. A *p*-value of $< 0.05$ was considered statistically significant. For the validation of calculated Hb data, the Spearman’s rho Correlation coefficient, and the Intraclass correlation coefficient, was utilised to ascertain the consistency of quantitative measurements and the within-subject variability of measured Hb between the two methods. Data analysis and graphical representations were performed using R version 4.0.0 software [22], and the RIs were computed using the “*referenceIntervals*” package version 1.2.0 [23].

Finally, for a more detailed evaluation of the impacts of captivity on nestling’s blood parameters, sample variance and the correlations between blood parameters were evaluated by Principal Component Analysis (PCA) using the “*factominer*” package [24]. This method visually presents associations between blood parameters with a factor map, providing a graphic representation of the statistical relationships among different categories of variables. The dimensions encapsulating the majority of the data variability are presented in the graph (dimensions 1 and 2), and the similarity or dissimilarity between captive-born and free-ranging nestlings is depicted as positions of the data from both populations, relative to the two dimensions. The Kaiser-Meyer-Olkin (KMO) measure of sampling adequacy was employed to assess the utility of the PCA.

## Results

### Data processing and reference population selection

The aim of this study was to establish RIs for Bonelli’s eagle employing an indirect approach [25]. The initial dataset encompassed 516 records and 47 variables. These data were cleansed, and in order to mitigate variability, selection and exclusion criteria were implemented, selecting a reference population of 117 nestlings and 49 adults. The data from wild adult eagles and juveniles (9 records each) was deemed unsuitable for use as a reference population due to the insufficient number of records (see Table 2 and Fig. 2).

When the effects of age and sex were assessed it was observed that only age significantly influenced the variation of several blood parameters, and no significant sex-based differences were discernible in nestlings or adults.

In contrast, captivity exhibited a significant impact on nestlings, as most parameters demonstrated prominent differences between wild and captive nestlings. This distinction was also validated by a PCA (as detailed below), therefore, data fulfilling all selection criteria were grouped by age and captivity status, and RIs were computed for three reference populations: wild nestlings ($n=72$), captive nestlings ($n=45$), and adults ($n=49$) (Fig. 2).

### Health parameters

Across all stages of development, Bonelli’s eagles displayed a clear sexual dimorphism with females being physically larger, resulting in consistently higher body weights in comparison to males, a pattern observed in both wild and captive nestlings (Table 3). However, there was no discernible impact of age, sex, or captivity status on other physiological parameters, such as body temperature, heart rate, or respiratory rate, showing no significant differences between both nestling and adult stages (Table 4).

**Table 3:** Weight (g) of healthy nestlings ($<50$ days of age) and adults ($>4$ years of age) of Bonelli’s eagles, categorized by sex

	Female mean (sd), median	n	Male mean (sd), median	n	Stat. significance^b^
**Nestling (captive)**	1824.0 (251.34), 1800	21	1409.54 (152.83), 1405	24	^***^
**Nestling (wild)**	1954.45(196.97), 2000	29	1566.72(217.6), 1595	40	^***^
**Adult (captive)**	2400.83 (296.72), 2350	23	1606.67 (419.07), 1650	27	^***^

Mean (sd) and number of reference individuals are provided for each group. Females consistently exhibit higher weight than males in all age groups. *T*-test significance *p*-values: $$p<0.001^{***}$$

**Table 4:** Temperature ($^{\circ }$C), heart rate in beats per minute (bpm), and respiratory rate in breaths per minute (bm) of healthy nestlings ($<50$ days of age) and adults ($>4$ years of age) of Bonelli’s eagles

	Nestlings		Adults	
	Mean (sd), median	n	Mean (sd), median	n
**Temperature (** $^{\circ }$**C)**	40.16 (0.79), 40.1	94	41.17(10.87), 42	49
**Heart rate (bpm)**	283.07(65.68), 266	83	262.5 (84.21), 254	49
**Respiratory rate (bm)**	50.51(16.01), 48	78	62.21(51.3), 44	49

Data were obtained during the physical examination at the time of handling. Mean (sd), median, and number of reference individuals are provided for each group. Neither sex nor age exerted any significant effect on any of these parameters

### Principal component analysis’s assessment of captivity effect

**Fig. 3:**
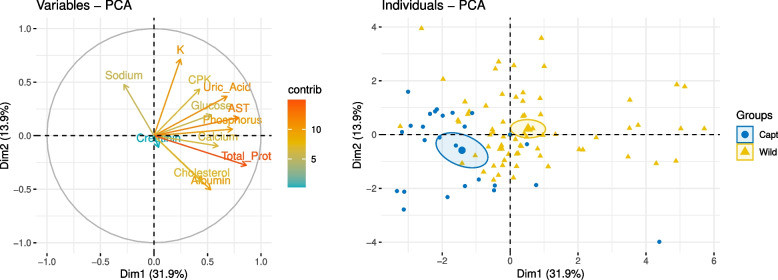
Principal Component Analysis (PCA) factor map displaying blood parameter data from the Bonelli’s eagle nestling reference population (<50 days of age) ($n=117$) (A) PCA factor map illustrates the relationships between biochemical parameters in the total population as coordinates in Dimension 1 and 2 (Dim1 and Dim2). The parameters contributing the most to each dimension are depicted by the longest distance to the coordinate origin and are color-coded with warmer colors (red and orange). Total Proteins and AST exert the highest contributions to Dim 1, while Potassium (K) has the highest contribution to Dim 2. (B) PCA biplot categorized by captivity. Individuals of captive (blue) and wild (yellow) birds are represented as coordinates in the first two-dimensional plane. The confidence ellipse delineates the region containing 95% of samples, indicating that the reference population of nestlings clusters into two distinct populations

A PCA was used to investigate values for various blood parameters derived from the nestling reference population (Fig. 3). This analysis enabled the identification of concealed patterns and correlations within our dataset and reduced the dataset’s dimensionality to two principal components (PC1 and PC2) while minimizing information loss. Our PCA had a Kaiser-Meyer-Olkin (KMO) measure of KMO= 0.53, indicative of its utility. The initial two dimensions accounted for 45.8% of the variation. The parameters showing highest contribution for dimension 1 were: TP and AST. For dimension 2 was K. Additionally, the biplot of individual data validated the results obtained with previous univariate test, showing two confidence ellipses that contain 95% of data with a distinct separation between samples from wild and captive nestlings that group in opposite sides of the biplot. This supported the calculation of separate RIs for these two distinct groups of nestlings within the reference population, according to origin or captivity (Fig. 2).

### Reference intervals for haematological and biochemical parameters

As indicated above, the reference population was segregated into three groups based on age and captivity status. Prior to the calculation of RIs, data for Hb obtained by the two methods (VetScan and calculated Hb) were validated. In our analysis, we found a Spearman’s rank correlation coefficient of *R*= 0.453, *p*= 0.00074, and an Intraclass correlation coefficient of ICC=0.67 between the two methods of measuring Hb. This substantial positive correlation suggests that there is a consistent trend of association of values obtained with both methods.

While no differences were observed between male and female birds, captivity exerted a significant effect on most blood parameters of nestlings. Therefore, the Lymphocyte count was lower in wild birds, resulting in a higher H/L ratio compared to captive birds (Tables 5 and 7). Similarly, other biochemical parameters, such as AST, CPK, Phos, K and UA were significantly higher in wild nestlings in comparison to captive ones (Tables 6 and 9).

**Table 5:** RI and descriptives of **haematological parameters** for the reference population of **free wild Bonelli’s eagle nestlings**

Parameter	Units	RI	mean (sd), median	min	max	n	Stat significance
**PCV**	%	27.0 - 38.77	31.3 (4.07), 31.0	20.0	45.0	67	
**RBC**	$10^{12}\;L^{-1}$	1.32 - 2.84	2.06 (0.38), 2.07	1.28	2.92	68	
**Hb**	$g\; dL^{-1}$	9.0 - 12.9	10.4 (1.4), 10.3	6.6	15.0	52	
**MCV**	*fl*	93.15 - 214.5	153.81 (30.95), 149.75	101.88	237.41	51	
**MCH**	*pg*	30.36 - 71.71	51.03 (10.55), 50.33	31.02	79.14	53	
**MCHC**	$g\; L^{-1}$	33.3 - 33.34	33.31 (0.05), 33.33	33.0	33.34	50	
**WBC**	$10^{9}\;L^{-1}$	4331.27 - 19545.46	11783.2 (3995.05), 12210	4180.0	20460.0	68	
**% Heterophils**	%	31.86 - 85.38	58.62 (13.65), 60.0	34.0	88.0	65	
**Heterophil count**	$10^{9}\;L^{-1}$	2714.4 - 16917.12	7038.42 (3166.55), 6252.4	2695.0	17463.6	65	
**% Lymphocytes**	%	10.0 - 45.78	21.75 (9.33), 20.0	10.0	46.0	65	
**Lymphocyte count**	$10^{9}\;L^{-1}$	374.88 - 6298.72	2362.48 (1567.56),1953.6	369.6	6406.0	65	a^***^
**% Eosinophils**	%	2.0 - 49.63	16.18 (11.38), 14.0	2.0	50.0	62	
**Eosinophil count**	$10^{9}\;L^{-1}$	211.75 - 8967.75	2102.45 (1885.88), 1744.6	206.8	9306.0	62	
**% Monocytes**	%	-	5.38 (3.47), 5.0	0.0	14.0	40	
**Monocyte count**	$10^{9}\;L^{-1}$	-	772.41 (801.85), 580.8	0.0	4637.6	40	
**% Basophils**	%	-	1.82 (1.24), 2.0	1.0	6.0	19	
**Basophil count**	$10^{9}\;L^{-1}$	-	423.92 (652.62), 184.8	75.0	2378.2	19	
**H/L ratio**		0.87 - 16.27	4.24 (3.35), 3.25	0.8	16.67	65	a^***^

Mean(sd), median, minimum and maximum values and number of reference samples used to determine RI (n) are included. In the case of a parameter containing too many zero values to calculate a RI, a range of observed values is presented. *PCV *haematocrit, *RBC *red blood cell count, *MCV *mean cell volume, *MCV *mean corpuscular volume, *MCH *mean corpuscular haemoglobin, *MCHC *mean corpuscular haemoglobin concentration, *WBC *white blood cell count, *H/L ratio *Heterophils/Lymphocytes ratio. Results of *U* Test (*p* value) a: Differences between wild and captive nestlings $p<0.001$^***^

**Table 6:** RI and descriptives of **biochemical parameters** for the reference population of **free wild Bonelli’s eagle nestlings**

Parameter	Units	xRI	mean(sd), median	min	max	n	Stat significance
**Alb**	$g\; L^{-1}$	12.7 - 21.8	18(2.4), 18.5	12.0	24.0	68	
**AST**	$U L^{-1}$	125.84 - 455.75	213.07(67.44), 202.0	119.8	518.6	70	a^***^
**Ca**	$mmol.L^{-1}$	2.28 - 3.49	2.77(0.4), 2.73	1.61	3.82	64	
**Cho**	$mmol.L^{-1}$	2.82 - 11.29	7.06(2.16), 6.53	3.75	12.24	36	
**CPK**	$U.L^{-1}$	1154.77 - 4930.45	3042.61(963.2), 2856.0	1377.0	5590.0	69	a^***^
**Crea**	$\mu mol.L^{-1}$	-	49.50(48.62), 30.94	0.88	155.58	14	
**Glu**	$mmol.L^{-1}$	11.57 - 25.91	16.79(3.16), 16.26	11.52	26.22	69	
**Phos**	$mmol.L^{-1}$	1.41 - 3.11	2.07(0.45), 2.02	1.18	3.95	68	a^**^
**K**	$mmol.L^{-1}$	3.16 - 9.52	5.54(1.84), 5.1	2.4	11.9	63	a^***^
**Na**	$mmol.L^{-1}$	138.52 - 162.38	148.31(11.45), 146.0	126.0	211.0	60	
**TP**	$g.L^{-1}$	22.6 - 61.4	38.3(10.1), 35.0	16.0	63.0	60	a^**^
**UA**	$\mu mol.L^{-1}$	243.87 - 1382.91	688.78(305.73), 618.59	243.87	1391.83	70	a^***^

Mean(sd), median, minimum and maximum values and number of reference samples used to determine RI (n) are included. When less than 20 reference samples were available, only descriptives are presented. In the case of a parameter containing too many zero values to calculate a RI, a range of observed values is presented. *Alb *Albumin, *AST *Aspartate aminotransferase, *Ca *Calcium, *Cho *Cholesterol, *CPK *Creatine phosphokinase, *Crea *Creatinine, *Glu *Glucose, *Phos *Phosphorus, *K *Potassium, *Na *Sodium, *TP *Total protein, and *UA *Uric Acid. Results of *U* Test (*p* value) a: Differences between wild and captive nestlings $p<0.001$^***^, $p<0.01$^**^

**Table 7:** RI and descriptives of **haematological parameters** for the reference population of Bonelli’s eagle **nestlings born in captivity**

Parameter	Units	RI	mean (sd), median	min	max	n	Stat significance
**PCV**	%	23.1 - 38.8	31.62 (5.16), 31.0	23.0	52.0	43	b^***^
**RBC**	$10^{12}\;L^{-1}$	1.02 - 2.96	1.99 (0.5), 1.98	1.04	3.46	45	
**Hb**	$g\; dL^{-1}$	7.62 - 12.3	10.09 (1.35), 10.33	7.60	13.33	45	b^***^
**MCV**	*fl*	92.2 - 224.61	159.86 (42.28), 155.96	83.82	269.23	27	b^*^
**MCH**	*pg*	31.97 - 80.32	53.05 (14.1), 51.16	27.95	89.71	27	b^**^
**MCHC**	$g\; L^{-1}$	33.3 - 33.35	33.16 (0.54), 33.32	30.4	33.34	23	
**WBC**	$10^{9}\;L^{-1}$	5701.56 - 19716.9	12709.23 (3575.41), 12980	4670.0	19800.0	45	
**% Heterophils**	%	39.39 - 67.94	51.66 (11.83), 52.0	25.0	83.0	36	
**Heterophil count**	$10^{9}\;L^{-1}$	1393.13 - 11006.12	6592.95 (2327.76), 6375.6	3341.8	13512.4	41	b^***^
**% Lymphocytes**	%	11.0 - 46.04	29.23 (9.73), 28.0	9.0	53.0	38	
**Lymphocyte count**	$10^{9}\;L^{-1}$	1122.0 - 7567.7	3825.55 (1830.35), 3375.0	1122.0	8162.0	41	a^***^
**% Eosinophils**	%	1.59 - 26.59	114.09 (6.38), 14.5	1.0	26.0	40	
**Eosinophil count**	$10^{9}\;L^{-1}$	107.8 - 3617.91	1794.49 (930.33), 1703.9	107.8	3823.6	37	b^***^
**% Monocytes**	%	1.0 - 9.17	4.55 (2.35), 4.0	1.0	9.0	39	
**Monocyte count**	$10^{9}\;L^{-1}$	68.2 - 1695.38	726.71 (471.95), 573.1	68.2	1738.0	39	
**% Basophils**	%	-	1.5 (0.71), 1.0	1.0	3.0	14	
**Basophil count**	$10^{9}\;L^{-1}$	-	198.88 (80.96), 180.4	97.0	323.4	14	b^***^
**H/L ratio**		0.39 - 3.19	2.21 (1.62), 1.86	0.57	7.78	36	a^***^; b^**^

Mean(sd), median, minimum and maximum values and number of reference samples used to determine RI (n) are included. When less than 20 reference samples were available, only descriptives are presented. *PCV *haematocrit, *RBC *red blood cell count, *MCV *mean cell volume, *MCH *mean corpuscular haemoglobin, *MCHC *mean corpuscular haemoglobin concentration, *WBC *white blood cell count, *H/L ratio *Heterophils/Lymphocytes ratio. Results of *U* Test (*p* value) a: Differences between wild and captive nestlings, b: Differences between nestlings and adults $$p< 0.001^{***}, p< 0.005^{**}, p< 0.05^{*}$$

**Table 8:** RI and descriptives of **haematological parameters** for the reference population of **adult Bonelli’s eagles**

Parameter	SI Units	RI	mean (sd), median	min	max	n	Stat significance
**PCV**	%	33.23 - 46.99	40.11 (3.51), 40.5	33.0	48.0	46	b^***^
**RBC**	$10^{12}\;L^{-1}$	1.37 - 2.84	2.1 (0.37), 2.09	1.25	2.86	45	
**Hb**	$g\; dL^{-1}$	10.64 - 15.52	13.08 (1.24), 13	10.6	16.33	47	b^***^
**MCV**	*fl*	145.24 - 265.48	189.95 (31.15), 183.17	132.87	269.66	47	b^*^
**MCH**	*pg*	48.41 - 108.96	76.23 (80.53), 63.76	44.3	660.75	45	b^**^
**MCHC**	$g\; L^{-1}$	32.49 - 34.18	34.0 (4.4), 33.33	28.18	42.90	43	
**WBC**	$10^{9}\;L^{-1}$	3617.48 - 16317.38	9967.43 (3239.83), 9456	4620.0	17600.0	48	
**% Heterophils**	%	44.5 - 76.88	63.13 (9.57), 64.0	31.00	77.00	44	
**Heterophil count**	$10^{9}\;L^{-1}$	1445.78 - 11557.092	6501.44 (2579.46), 6441.6	2217.6	13170.04	44	b^***^
**% Lymphocytes**	%	16.0 - 53.1	26.55 (8.44), 24.5	16.0	54.00	42	
**Lymphocyte count**	$10^{9}\;L^{-1}$	1022.87 - 6104.08	2672.14 (1157.25), 2468.95	1016.4	6142.0	42	
**% Eosinophils**	%	3.0 - 14.8	6.6 (3.02), 6.0	3.00	15.00	43	
**Eosinophil count**	$10^{9}\;L^{-1}$	190.24 - 1742.4	658.65 (358.06), 600.6	189.06	1768.8	43	b^***^
**% Monocytes**	%	-	3.87 (2.34), 4.0	0.0	9.0	48	
**Monocyte count**	$10^{9}\;L^{-1}$	-	381.78 (264.93), 345.48	0.0	1016.4	48	
**% Basophils**	%	-	0.45 (0.92), 0.0	0.0	4.0	48	
**Basophil count**	$10^{9}\;L^{-1}$	-	48.78 (107.1), 0.0	0.0	563.2	48	b^***^
**H/L ratio**		0.08 - 0.59	0.28 (0.15), 0.23	0.08	0.75	33	b^**^

Mean (sd) median, minimum and maximum values and number of reference samples used to determine RI (n) are included. *PCV *haematocrit, *RBC *red blood cell count, *MCV *mean cell volume, *MCV *mean corpuscular volume, *MCH *mean corpuscular haemoglobin, *MCHC *mean corpuscular haemoglobin concentration, *WBC *white blood cell count, *H/L ratio *Heterophils/LYMs ratio. Results of *U* Test (*p* value) b: Differences between nestlings and adults $$p< 0.001^{***}, p< 0.005^{**}, p< 0.05^{*}$$

When evaluating age effects among captive birds, age significantly influenced the majority of blood parameters in nestlings and adults. Notably, captive nestlings demonstrated significantly lower levels of PCV, Hb, MCV compared to adult eagles and Heterophil levels compared to adult eagles, whereas Eosinophils and Basophils were significantly elevated (Tables 7 and 8). Regarding biochemical parameters, nestlings displayed significantly elevated CPK, Phos, and K levels, while the adult population exhibited increased AST, CPK and GLU levels (Tables 9 and 10). In adult birds, CPK values were significantly higher in males than females (Table 10).

**Table 9:** RI and descriptives of **biochemical parameters** for the reference population of Bonelli’s eagle **nestlings born in captivity**

Parameter	Units	RI	mean (sd), median	min	max	n	Stat significance
**Alb**	$g\; L^{-1}$	6.6 - 24.2	15.9 (3.9), 14.5	11.0	24.0	24	
**AST**	$U.L^{-1}$	80.65 - 212.59	153.93 (31.15), 148.4	113.0	255.2	24	a^***^; b^***^
**Ca**	$mmol.L^{-1}$	2.14 - 3.24	2.71 (0.46), 2.71	1.41	3.72	21	
**Cho**	$mmol.L^{-1}$	-	8.49 (5.98), 6.54	5.16	28.35	14	
**CPK**	$U.L^{-1}$	275.97 - 3355.53	1815.75 (785.62), 1775.5	376.0	3082.0	24	a^***^; b^***^
**Crea**	$\mu mol.L^{-1}$	-	82.21 (70.72), 76.02	7.07	191.83	6	
**Glu**	$mmol.L^{-1}$	12.42 - 20.35	16.49 (2.88), 16.38	10.44	24.78	22	b^***^
**Phos**	$mmol.L^{-1}$	1.29 - 2.32	1.6 (0.57), 1.75	0.06	2.31	19	a^**^
**K**	$mmol.L^{-1}$	0.99 - 6.82	3.91 (1.49), 3.6	1.8	6.6	23	a^***^
**Na**	$mmol.L^{-1}$	124.9 - 182.62	154.48 (13.45), 153	138.0	183.0	23	
**TP**	$g.L^{-1}$	-	31.5 (7.9), 31.0	19.0	52.0	15	a^**^
**UA**	$\mu mol.L^{-1}$	71.38 - 645.95	306.32 (176.66), 240.89	71.38	713.76	24	a^***^

Mean(sd), median, minimum and maximum values and number of reference samples used to determine RI (n) are included. When less than 20 reference samples were available, only descriptives are presented. In the case of a parameter containing too many zero values to calculate a RI, a range of observed values is presented. *Alb *Albumin, *AST *Aspartate aminotransferase, *Ca *Calcium, *Cho *Cholesterol, *CPK *Creatine phosphokinase, *Crea *Creatinine, *Glu *Glucose, *Phos *Phosphorus, *K *Potassium, *Na *Sodium, *TP *Total protein, and *UA *Uric Acid. a: Results of *U* Test (*p* value) a: Differences between wild and captive nestlings, b: Differences between nestlings and adults $$p< 0.001^{***}, p< 0.01^{**}$$

**Table 10:** RI and descriptives of **biochemical parameters** for the reference population of **adult Bonelli’s** eagles

Parameter	Units	RI	mean(sd), median	min	max	n	Stat significance
**Alb**	$g\; L^{-1}$	12.20 - 24.0	18.10 (3.0), 18.0	12.0	27.0	49	
**AST**	$U.L^{-1}$	121.8 - 300.15	187.93 (50.29), 179.7	110.0	363.0	48	b^***^
**Ca**	$mmol.L^{-1}$	2.40 - 3.21	2.7 (0.30), 2.63	2.0	4.10	49	
**CPK (females)**	$U.L^{-1}$	226.0 - 1255.03	579.19 (335.47), 474	226.0	1734	26	b^***^
**CPK (males)**	$U.L^{-1}$	171.14 - 1325.05	855.72 (305.94), 766	466.0	1594.0	23	b^***^
**Glu**	$\mu mol.L^{-1}$	12.65 - 22.59	17.62 (2.54), 17.65	12.38	23.87	49	b^***^
**Phos**	$\mu mol.L^{-1}$	0.32 - 1.92	1.05 (0.44), 1.04	0.32	2.08	49	
**K**	$mmol.L^{-1}$	1.50 - 6.80	3.1 (1.33), 2.80	1.50	6.80	49	
**Na**	$mmol.L^{-1}$	138.65 - 183.60	157.25 (11.87), 152.0	137.0	188.0	49	
**TP**	$g.L^{-1}$	32.0 - 48.0	38.9 (5.0), 38.50	31.0	63.0	49	
**UA**	$\mu mol.L^{-1}$	199.85 - 1067.67	449.67(194.50), 404.46	196.28	1136.07	22	

Mean(sd), median, minimum and maximum values and number of reference samples used to determine RI (n) are included. When less than 20 reference samples were available, only descriptives are presented. *Alb *Albumin, *AST *Aspartate aminotransferase, *Ca *Calcium, *Cho *Cholesterol, *CPK *Creatine phosphokinase, *Crea *Creatinine, *Glu *Glucose, *Phos *Phosphorus, *K *Potassium, *Na *Sodium, *TP *Total protein, and *UA *Uric Acid. Results of *U* Test (*p* value): b: Differences between nestlings and adults $$p<0.001^{***}$$

### Detection of lead in blood

During the analysis of blood Pb concentrations in both wild and captive nestlings using the LeadCare II System$^{\copyright }$, the majority of results were either extremely low or beneath the detection limit (data not shown). However, due to its heightened sensitivity, ICP-MS analysis uncovered a significantly elevated Pb concentration in the blood of wild nestlings in comparison to captive birds, while for most captive nestlings, Pb levels were proximate to the detection limit (Table 11).

**Table 11:** Descriptives of Pb ($\mu$g/dL) detected in blood from the reference population of wild and captive Bonelli’s eagle nestlings

Status	mean (sd), median	min	max	n	Stat significance
**Wild**	1.73 (1.87), 1.02	0.006	8.12	50	^***^
**Captive**	0.035 (0.11), 0.006	0.006	0.44	15	^***^

The mean value (sd) and number of reference samples used (n) are presented. Significant differences have been found between captive and wild birds (*t * Test $$p< 0.001^{***}$$)

## Discussion

In the present study, we successfully established haematological and biochemical reference values for wild and captive nestlings, as well as adults of the Bonelli’s eagle. Moreover, comprehending baseline Pb concentrations is of particular importance, given that susceptibility appears to vary across species [16]. To the authors’ understanding, this is the first study determining reliable RIs for this endangered species in alignment with the ASVCP recommendations. Our study extensively complements the data previously reported for the Bonelli’s eagle [15]. Numerous factors corroborate this claim. On one hand, our analysis was predicated on an extensive and meticulously collated dataset ($n=516$). This dataset comprised in-depth analytical reports from a seven-year longitudinal study, encapsulating data from both wild and captive avian populations including different age groups. On the second hand, we implemented a standardised protocol to define a representative reference population. Using data mining methods and applying a comprehensive set of exclusion criteria, we selected reports of confirmed healthy individuals, reducing potential biological and methodological variations. Lastly, we employed robust statistical tools tailored specifically to uphold the ASVCP standards during the RI calculation, such as the “*factominer*” and “*referenceIntervals*” packages [23, 24]. This provided the obtained data with a substantial level of robustness and reliability. For many raptor species and other wild birds, it is particularly important to exclude potential sources of blood parameter variability, attributable to physiological or biological factors such as reproductive status at the time of sampling, handling stress, or laboratory management [1–3, 12].

Our study revealed that Bonelli’s eagle haematological parameters show no significant sex-based variations. This observation is consistent with findings for various raptors, including peregrine falcons [26], cooper hawks [5], american kestrels [27], marsh harriers [28, 29], golden eagle [30], harpy eagle [31], Montagu’s harriers [32], and sharp-shinned hawks [33]. Contrastingly, species-specific differences exist, as illustrated by the chimango caracaras, which exhibit sex-dependent variations in eosinophil counts [34].

In various bird species, the increase in hematocrit and RBC (erythropoiesis) with age is well-documented as a natural physiological process. Consequently, nestlings generally display lower levels of PCV, RBC, Hb, and MCV compared to adults. Moreover, in several raptor species, Hb levels have been shown to be particularly affected by age, showing a significant increase as development progresses, until they reach adulthood [35–37].

The reduced number of heterophils observed in captive nestlings can be attributed to the incomplete maturation of hematopoiesis, a process that is fully developed in adult birds, as described by [38]. Additionally, in avian species, eosinophils are hypothesized to act as early modulators of inflammation, whereas basophils primarily contribute to immediate immune responses, a role distinct from their function in mammals, to our knowledge, this cells have not been reported to be affected by age in other wild raptors (reviewed in [38]).

Previous studies have indicated that nestling survival in wild populations is associated with lower counts of specific blood cell types, including heterophils, eosinophils, and the proportion of lymphocytes, with an interaction involving the H/L ratio [39]. Our findings demonstrate a significantly higher H/L ratio in wild nestlings, a reliable indicator of stress and overall health in wild birds. It has been shown to predict stress [40] and long-term survival in birds [41] and it is influenced, not only by various ecological and physiological factors [42] but also by physical condition, sex, and genetic diversity [43]. While H/L ratio and circulating corticosterone are both stress indicators, they may respond differently to various stressors and should not be used interchangeably [44]. Therefore, despite measures taken to minimize handling stress, including the use of experienced handlers and skilled veterinarians, our results suggest that handling or other environmental stressors may still influence the H/L ratio in wild nestlings, even in otherwise healthy populations.

Similar to the findings for haematological parameters, most biochemical markers in our study did not exhibit sex-dependent variation. This observation contrasts with results from White-tailed Sea Eagle nestlings, where marked sex differences were detected [45]. However, in agreement with other species, age significantly influenced several biochemical parameters, including AST and CPK. The reference intervals determined for these markers align with those reported in other wild raptors, such as the Bald Eagle [46], Booted Eagle [47], and Imperial Eagle [48]. More importantly, a previous study on wild Bonelli’s Eagle nestlings [15] provided certain plasma chemistry values that overlap with our findings; nevertheless, our analysis encompassed a more extensive reference population (117 nestlings versus 28) and additionally included 49 adults, enabling a more comprehensive evaluation of developmental changes in blood parameters.

Age has been shown to affect blood parameters in numerous avian species. For instance, in wild Scarlet Macaws, multiple blood biochemical values (Alb, AST, globulin, Glu, TP, Na, UA) increase with age, whereas others (CPK, Phos, K) decrease [49]. In nocturnal raptors, Phos, Ca, and alkaline phosphatase decline with age, while Na and $\gamma$-globulins increase [50]. Conversely, Egyptian Vulture nestlings exhibit higher concentrations of Crea, urate, Ca, Phos, and alkaline phosphatase than adults [51], underscoring the interspecific variability in avian blood parameters.

Our findings highlight marked disparities in several biochemical markers between wild and captive nestlings. Notably, the PCA analysis identified AST, K, and TP as elevated in wild nestlings, whereas Phos was higher in captive ones, underlining the distinction between the two populations based on captivity.

Research on blood parameters in wild birds reveals significant interspecific variation, and these differences are further influenced by captivity. For example, in Egyptian vultures, captive birds displayed elevated levels of TP, Alb, and AST, which may reflect differences in their physiological state compared to wild counterparts [51], while in another study, free-living condors had significantly lower TP, Alb, globulin, and magnesium levels compared to captive condors [52]. Moreover, it has been suggested that in captive birds, blood parameteres are influenced by various factors like physiological state, age, sex, nutrition, and circadian rhythm. Therefore, it is essential to account for these variability when interpreting data from captive populations [53].

In addition, CPK activity is particularly high in muscle tissue relative to the liver or kidneys, thereby serving as a reliable marker for muscle damage [54]. Consequently, elevated levels of CPK and AST have been observed in captured birds, suggesting muscle injury as a response to handling or other stressors [55]. Beyond this, alterations in these biochemical parameters such as CPK, AST may not only result from the stress associated with handling but also from other factors, including increased physiological demands [56]. In addition, UA can be affected by postprandial effects and dehydration [57]. Importantly, such conditions may go undetected during routine examinations of wild chicks, underscoring the complexity of interpreting these parameters.

Changes in these blood parameters, potentially tied to factors like stress, diet, or environmental conditions, require further investigation, especially when assessing both captive and wild eagles.

With regard to Pb determination, it is important to note that these data originate from samples of physiologically optimal birds and exhibit an asymmetrical distribution. The highest Pb concentrations were observed in a small number of wild individuals, while the majority displayed very low levels. Notably, none of the wild nestlings had blood Pb concentrations exceeding the threshold associated with overt clinical signs, as reported in previous studies [58–60]. Furthermore, no correlation was detected between Pb levels and the blood parameters studied in the reference population, suggesting that the observed Pb levels did not have a detectable physiological impact under the measured parameters.

While Pb levels serve as indicators of environmental pollution [61], there is no clear evidence of how these low concentrations might affect the future health of the nestlings. Previous research has reported Pb levels below 1.93 $\mu$g/dL in healthy Bonelli’s eagles, whereas individuals admitted to wildlife rescue centers exhibited levels exceeding 20 $\mu$g/dL [62]. Although this study included only a limited sample size ($n=6$ wild and $n=3$ captive birds), it supports the notion that low Pb concentrations may not result in overt clinical signs in this species. Similarly, a more recent study using anodic stripping voltammetry found no correlation between Pb levels and hematological or biochemical values in a population of 54 Bonelli’s eagles [63].

In the present study, only 6 wild nestlings (12% of the wild reference population) had Pb levels exceeding 2.5 $\mu$g/dL, a threshold previously associated with potential deleterious effects in golden eagles [64]. Upon re-examining the data for these individuals, we found that their hematological and biochemical parameters remained within the established reference intervals. However, other subclinical effects, such as behavioral changes or subtle neurological impairments, cannot be ruled out based on these data alone. Consequently, further research is necessary to understand how background Pb exposure might affect the development of Bonelli’s eagle nestlings in the wild.

## Conclusions

We have successfully established new Reference Intervals (RI) for both wild and captive nestlings of the Bonelli’s eagle, adhering to the guidelines set forth by the ASVCP.

Our research identified distinct differences in several blood parameters between populations of wild and captive-born birds. Specifically, wild nestlings exhibited significantly higher blood lead (Pb) levels compared to captive nestlings, even though these levels were not associated with pathological conditions. From a One Health perspective, the quantification of lead in the blood of wild nestlings serves as an effective sentinel, reflecting the ecosystem’s level of this pollutant.

The methodology we employed for calculating robust ranges, which prioritizes high-quality standards, comprehensive data sourcing, and carefully defined reference populations, offers a significant improvement over existing data. We believe this should gradually replace the current data, providing a reliable reference for researchers interested in this field.

## Data Availability

The data that support the findings of this study are available from GREFA WRC but restrictions apply to the availability of these data, which were used under license for the current study, and so are not publicly available. Data are however available from the authors upon reasonable request and with permission of GREFA.

## Abbreviations

Alb: Albumin
ASVCP: American Society for Veterinary Clinical Pathology
AST: Aspartate aminotransferase
Ca: Calcium
Cho: Cholesterol
CPK: Creatine phosphokinase
Crea: Creatinine
ELP: European Life Project
Glu: Glucose
GREFA: Grupo de Rehabilitación de la Fauna Autóctona y su Hábitat
Hb: Haemoglobin concentration
H/L: Heterophil to Lymphocyte ratio
K: Potassium
LYM: Lymphocyte count
MCH: Mean corpuscular haemoglobin
MCHC: Mean corpuscular haemoglobin concentration
MCV: Mean corpuscular volume
MON: Monocyte count
Na: Sodium
PCA: Principal Component Analysis
PCV: Haematocrit
Phos: Phosphorus
PLT: Platelet count
RBC: Red blood cell count
RI: Reference Intervals
sd: Standard deviation
TP: Total protein
UA: Uric Acid
WBC: White blood cell count
WRC: Wildlife Rehabilitation Centre
